# Random forest classification as a tool in epidemiological modelling: Identification of farm-specific characteristics relevant for the occurrence of *Fasciola hepatica* on German dairy farms

**DOI:** 10.1371/journal.pone.0296093

**Published:** 2023-12-21

**Authors:** Andreas W. Oehm, Yury Zablotski, Amely Campe, Martina Hoedemaker, Christina Strube, Andrea Springer, Daniela Jordan, Gabriela Knubben-Schweizer

**Affiliations:** 1 Institute of Parasitology, Vetsuisse Faculty, University of Zurich, Zurich, Switzerland; 2 Clinic for Ruminants with Ambulatory and Herd Health Services, Ludwig-Maximilians-Universität Munich, Oberschleissheim, Germany; 3 Department of Biometry, Epidemiology and Information Processing, WHO Collaborating Center for Research and Training for Health at the Human-Animal-Environment Interface, University of Veterinary Medicine, Foundation, Hannover, Germany; 4 Clinic for Cattle, University of Veterinary Medicine, Foundation, Hannover, Germany; 5 Institute for Parasitology, Centre for Infection Medicine, University of Veterinary Medicine Hannover, Hannover, Germany; University of Illinois College of Veterinary Medicine, UNITED STATES

## Abstract

*Fasciola hepatica* is an internal parasite of both human and veterinary relevance. In order to control fasciolosis, a multitude of attempts to predict the risk of infection such as risk maps or forecasting models have been developed. These attempts mainly focused on the influence of geo-climatic and meteorological features. Predicting bovine fasciolosis on farm level taking into account farm-specific settings yet remains challenging. In the present study, a new methodology for this purpose, a data-driven machine learning approach using a random forest classification algorithm was applied to a cross-sectional data set of farm characteristics, management regimes, and farmer aspects within two structurally different dairying regions in Germany in order to identify factors relevant for the occurrence of *F*. *hepatica* that could predict farm-level bulk tank milk positivity. The resulting models identified farm-specific key aspects in regard to the presence of *F*. *hepatica*. In study region North, farm-level production parameters (farm-level milk yield, farm-level milk fat, farm-level milk protein), leg hygiene, body condition (prevalence of overconditioned and underconditioned cows, respectively) and pasture access were identified as features relevant in regard to farm-level *F*. *hepatica* positivity. In study region South, pasture access together with farm-level lameness prevalence, farm-level prevalence of hock lesions, herd size, parity, and farm-level milk fat appeared to be important covariates. The stratification of the analysis by study region allows for the extrapolation of the results to similar settings of dairy husbandry. The local, region-specific modelling of *F*. *hepatica* presence in this work contributes to the understanding of on-farm aspects of *F*. *hepatica* appearance. The applied technique represents a novel approach in this context to model epidemiological data on fasciolosis which allows for the identification of farms at risk and together with additional findings in regard to the epidemiology of fasciolosis, can facilitate risk assessment and deepen our understanding of on-farm drivers of the occurrence of *F*. *hepatica*.

## Introduction

Parasitic infections are complex in their nature and a threat for host health and well-being. Globally, *Fasciola hepatica* is an internal parasite of both human and veterinary relevance. Between 2.4 million and 17 million people are estimated to be infected with liver flukes and more than 180 million are assumed to be at risk [1–3]. Ruminant livestock represent a critical reservoir for human infection in some geographical areas [4–6]. In Europe, farm-level prevalence of fasciolosis in dairy cows has been determined to regionally range from 7% to 80% [7–9]. *Fasciola hepatica* has an intricate lifecycle that incorporates intermediate snail and definitive mammal hosts. Eggs shed in the feces of the mammalian host release miracidia in fresh water which subsequently infect susceptible amphibious snails of the *Lymnaeidae* family. The intermediate host holds a key position in transmission and asexual reproduction of *F*. *hepatica* [10–12]. Its distribution is largely dependent on geo-climatic, ecological, anthropogenic, and habitat-associated factors. Even though several mathematical modelling approaches to predict the occurrence of fasciolosis have been presented, the spatial distribution of *F*. *hepatica* remains incompletely understood, especially on farm level [13–15]. Recent efforts have focused on quantifying and understanding the relevance of climatic and meteorological conditions to characterise the occurrence of *F*. *hepatica* [14, 16, 17]. As an example, Roessler et al. [14] have incorporated meteorological and temperature-related variables as well as information on local soil and vegetation properties to predict the occurrence of the intermediate host. However, farm conditions and individual farm-related epidemiological factors hold an important share in promoting transmission on a regional scale and models solely based on climatic and environmental factors are only partly able to predict the presence of *F*. *hepatica* on a certain farm [18–20]. Besides the need to improve our understanding of the spatial distribution and the relevance of environmental (e.g. vegetation and soil characteristics) and climatic (e.g. temperature, humidity, rainfall) aspects in *F*. *hepatica* transmission, research is required to predict the presence of *F*. *hepatica* on farm level based on farm characteristics, management practices, and production parameters [13, 21, 22]. A comprehensive knowledge of relevant aspects of this biological system, their interplay, and common occurrence is paramount in order to fully comprehend the on-farm presence of *F*. *hepatica*. Data-driven machine learning approaches recently have drawn attention since they have the capability to determine important aspects and patterns in epidemiological situations [23–25]. Random forest (RF) is a machine learning approach that allows for the identification of relevant features in data sets despite the potential presence of interactions and correlations among variables. Furthermore, RF is known for its inherent feature of variable importance ranking [24, 26]. This creates a suitable prerequisite for implementation of RF in modelling of parasitic infections and for identifying key farm features associated with parasite presence. The aim of this work was the application of a RF approach to a multifactorial data set of dairy cow housing conditions, management practices, and production parameters to identify and rank relevant covariates for farm-level presence of *F*. *hepatica*. For this purpose, we were able to build upon previous work of our group using a cross-sectional data set [27–29]. The application of a machine learning approach increases the understanding of the on-farm network of factors affecting farm-level positivity for this important helminth. Furthermore, describing farm-level occurrence of *F*. *hepatica* based on farm characteristics contributes to our knowledge of this system and provides a set of relevant aspects that are to be considered in regard to *F*. *hepatica* in an applied epidemiological context.

## Materials and methods

### Study population

Data on housing conditions, management regimes, and animal health on German dairy farms were collected in the context of an extensive, descriptive, cross-sectional study [30]. Three geographically and structurally different dairying regions (region North: federal states of Lower Saxony and Schleswig-Holstein; region East: federal states of Thuringia, Saxony-Anhalt, Brandenburg, and Mecklenburg-Western Pomerania; region South: federal state of Bavaria) were included in order to cover the range of potential animal husbandry practices in dairy production. A total number of 86,304 dairy cows (North: 24,980 cows; East: 49,936 cows South: 11,388 cows) on 765 farms (North: 253; East: 252; South: 260) were included in the study. Sample size calculation and farm selection are described in [28, 29, 31]. In brief, sample size calculation was based on the formula suggested for prevalence studies:

$$n=\frac{Z^{2}P(1-P)}{d^{2}}$$

where *n* is the sample size to be calculated, *Z* the level of confidence, *P* the assumed prevalence, and *d* the precision. Sample size was conceived to cover different distribution scenarios, i.e. different expected prevalences e.g. of *F*. *hepatica*. These different scenarios were calculated at a power of 80%, a significance level of 5%, and a confidence level of 95% in order to obtain an optimal and feasible sample size. A standard deviation of 7 was assumed [32, 33]. To be able to estimate the expected value with a certain degree of precision, a precision of ±1, ±2, ±3, and ±4 was used [33]. Given the aforementioned considerations and taking into account feasibility, the goal was to visit 250 farms within each of the three regions. Selection was stratified by administrative district of the farms and herd size, i.e. number of lactating and dry cows, within the federal states and study regions. Information for sampling was extracted from the national animal information data base (HIT) as well as from the Milchprüfring Bayern e.V. (Bavarian Milk Testing Association) and an automated approach yielded farms that were to be contacted. Selected farms received an invitation to participate as well as information on the study by mail. Interested farm managers had to autonomously contact their respective regional study team in order to arrange time and date of the farm visit. Within each region, 1,250 farms, i.e. five times the number of farms required to meet the sample size, were contacted in order to cover a response rate of 20%. Since participation rate turned out to be by far less than expected (<10%), a second round of invitations was sent out. Written consent for participation and data inspection was obtained from each farm. All data were handled in alignment with German and European data protection legislation. Farm visits were scheduled as one time assessments between January 2017 and August 2019.

### Data collection

Paper-based data entry forms and questionnaires were used to record data during the farm visit. Subsequently, all assessments were transferred to one SQL data base containing all the available information on the included animals and farms. All lactating and dry cows present on the day of the farm visit were included and from each animal, the individual ear tag identification number was recorded. Body condition score (BCS) was assessed using the system provided by Edmonson et al. [34]. Leg and udder cleanliness were recorded according to Cook and Reinemann [35]: 1 = little to no manure, 2 = minor splashings, 3 = plaques of manure, 4 = solid, confluent plaques of manure. Tarsal areas were assessed from a caudolateral perspective to detect any form of alterations [36, 37]. If hocks were covered by solid plaques of manure and hence an assessment was not possible, cows received a score of 6. Only the more severe alteration was documented. The Sprecher lameness scoring system [38] was implemented to record locomotion in loose housing facilities whereas the Stall Lameness Score (SLS) [39] was used to document weight shifting between the rear limbs, sparing of a limb while standing, unequal weight bearing when stepping from side to side, and standing on the edge of the kerb. Alterations of the tail were assessed as follows: 1 = no visible change, 2 = visible deviance of axis or bulge/swelling, 3 = amputated tail.

Production data on milk yield (in kg), milk fat (in kg), milk protein (in kg), as well as calving intervals (in days) were extracted from HIT and the national milk recording system (DHI). Production data were available on farm level, i. e. farm level milk yield, milk fat, and milk protein, respectively, adjusted for number of cows per farm for the three years prior to the farm visit. Calving intervals were available on cow level for the three years period prior as well. Information on somatic cell count (SCC) was available on cow level with monthly assessments for up to 12 months prior to the farm visit. Parity data were retrieved from DHI. A face-to-face, pen-and-pencil interview was conducted with the responsible farm manager as elaborated on by Jensen et al. [40]. In brief, the attitude of the farmers towards their work on the farm was assessed and farmers rated their consent to each statement on a five-point Likert scale (“strongly disagree”, “disagree”, “neutral”, “agree”, “strongly agree”). Moreover, management procedures on the animals during the period around calving were recorded as follows: 1 = in all/most cases; 2 = in suspicious cases, 3 = rarely/never. All attitude and management-related questions and the respective variables are compiled S2 Table. Pasture access for dairy cows, farming type (organic vs. conventional), and income type of the farm (dairy farming as main source of income vs. dairy farming as sideline source of income) were recorded during the interview as well. A bulk tank milk (BTM) sample was collected from the central bulk tank on each farm by the farm manager to be analysed for *F*. *hepatica* antibodies. Farm managers were asked to collect the sample towards the end of the grazing season (August–November) in order to increase comparability across farms. BTM antibodies were determined using the IDEXX Fasciolosis Verification Test (IDEXX GmbH) as previously reported [27]. ELISA results with a sample/positive control ratio (S/P) > 30% were considered seropositive.

### Data handling

Plausibility checks were run automatically within the central data base in alignment with a priori determined threshold values. Four of the co-authors carried out additional plausibility checks of all variables within the data set. In case of implausible values, they were checked for in the data base as well as in the original paper-based questionnaires and data entry forms to detect irregularities both during data export as well as during transcription of the written records. If implausible values could not be corrected based on the available sources, the record was set to “missing”. The statistical software R version 4.2.0 [41] was used for all statistical analyses. All implemented packages including references are summarised in S3 Table.

Body condition score, stratified by stage of lactation and breed, was categorised into undercondition, optimal condition, and overcondition according to previous work [31, 42, 43]. Leg and udder cleanliness were dichotomised into no/slight contamination (scores 1 and 2) and considerable contamination (scores 3 and 4). Likewise, hock lesions were dichotomised into no lesions and hairless spots vs. more severe lesions (swelling and/or wound). Cows in loose housing were classified as lame with a locomotion score ≥ 3 [44]. In tied cows, lameness was defined as the presence of at least two of the four behavioural patterns of the SLS during a 90 s observation period [39, 45, 46]. Tail changes were dichotomised into no changes (score 1) and visible changes (scores 2 and 3). All animal-level information on the aforementioned variables was raised to farm level by calculating farm-level prevalences.

A Bayesian bootstrap approach was conducted to obtain a single median value for the four available values for milk yield, milk fat, milk protein, and calving interval. As for SCC, information was available on cow level with up to twelve potential values. This approach enabled us to condense the available information for every single animal into one median value reflecting the individual cow. A second round of bootstrapping raised the information to the farm level. Parity was directly raised to farm level.

The five-point Likert scale items of the attitude variables were condensed from five to three categories, i.e. disagreement, neutrality, and agreement. As missing data were present in the scoring part of the data set (S1 Table) as well as in the attitude part and among the variables reflecting management procedures, a non-parametric multivariate imputation by chained random forest was implemented to impute the missing observations and replace them with plausible values [47]. This approach is able to substitute missing data using all other variables in the data set as predictors by combining random forest imputation and multivariate imputation by chained equations. Iterations are imputed for every single missing value multiple times until the Out-of-Bag (OOB) prediction error stops to improve i.e., the highest possible prediction accuracy is achieved. This allows for a realistic, plausible imputation and adheres to the original structure of the underlying data [47]. To complement the analyses on the imputed data and to allow for a direct comparison of a model using imputed data and a model based on a data set without missing values, we created a complete cases data set for each study region where observations with missing values for single variables were excluded from further analyses. A binary variable (*F*. *hepatica* seropositive/seronegative) was created based on the thresholds of the BTM ELISA: results with a sample/positive control ratio (S/P) > 30% were considered seropositive. *Fasciola hepatica* presence on a farm was defined as BTM seropositivity of a farm.

### Random forest for the identification and ranking of farm-level aspects important for the presence of *F*. *hepatica*

Breiman’s random forest algorithm for classification was applied to forecast *F*. *hepatica* presence on farms based on the set of covariates and to identify relevant key predictors [26]. The randomForest function [48] was implemented and tuneRF() identified the optimal tuning parameters i.e., mtry (= the number of variables evaluated at each node) and searched for the maximised prediction accuracy with respect to the OOB error estimate, i.e. identified the mtry value that produced the smallest OOB error. Each tree within the RF is constructed based on a random, varying bootstrap sample of the original data [26, 49]. Each node of every single tree is split in alignment with the best split among a random subset of all predictors. This procedure allows to address correlation from individual trees and is robust to overfitting. Moreover, at each iteration of the bootstrap, observations not included in the bootstrap are called OOB sample from which the OOB error estimate can be obtained by aggregation of all OOB predictions. The estimation of an unbiased estimate of the test set error hence is conducted internally during the run [50–52]. The data set was split into training and test data with a ratio of 70:30 of training vs. testing. A RF was generated on the training data and predictions were validated on the test data. Based on the obtained confusion matrix, evaluation metrics, i.e. precision, predictive accuracy, recall, and F1 score, were calculated. A total number of 1,000 trees was used at the tuning step. The importance of each of the covariates was assessed via the mean decrease accuracy (MDA), a means to indicate how much removing a single variable reduces the accuracy of the prediction. Accordingly, high ranking variables contribute the most to model fit and prediction accuracy in comparison with low ranking variables [26]. Permutation p-values were estimated for the random forest importance metrics of the included variables using the R package rfPermute [53]. A total number of 1,000 permutation replicates were run.

### Data accessibility

The final imputed data sets used for the current analyses are available in Mendeley Data at https://doi.org/10.17632/5p8tzvw9mh.1. The complete cases data sets are provided as supporting information (S1, S2 Data).

## Results

Study regions North and South were included in this work, since only two farms were positive for *F*. *hepatica* in region East. Parts of the descriptive data have been described elsewhere [27–29].

### Region North

A complete descriptive overview of continuous and categorical variables within the imputed data set is provided in Tables 1 and 2, respectively.

**Table 1:** Descriptive statistics of continuous variables within the imputed data set for each of the two study regions (North = 188 farms, South = 212 farms).

	North					South				
Variable	Mean	Range	1^st^ Qu.	Median	3^rd^ Qu.	Mean	Range	1^st^ Qu.	Median	3^rd^ Qu.
Underconditioned^1^	20.1	0.0–66.7	12.2	19.9	27.4	10.8	0.0–61.8	4.0	8.3	14.3
Optimally conditioned^2^	69.0	0.0–91.9	64.7	69.7	74.8	71.5	35.3–100.0	62.6	71.7	80.8
Overconditioned^3^	11.0	0.0–100.	3.8	7.7	13.8	17.7	0.0–60.7	7.9	15.7	25.1
Parity^4^	2.5	1.6–3.7	2.3	2.4	2.7	2.5	1.7–3.7	2.3	2.5	2.7
Udder hygiene^5^	22.7	0.0–71.1	12.5	19.0	30.0	21.4	0.0–76.3	9.7	19.3	29.4
Leg hygiene^6^	41.6	5.8–85.9	26.6	38.9	57.5	32.4	0.0–88.5	18.7	29.8	42.9
Hock lesions^7^	14.2	0.0–43.8	6.4	11.4	19.4	15.9	0.0–77.8	6.2	12.5	23.8
Lameness^8^	25.9	0.0–76.9	14.3	25.9	35.4	24.7	0.0–67.6	14.5	23.1	33.3
Tail changes^9^	11.7	0.0–42.3	6.6	10.1	14.6	5.8	0.0–28.9	2.1	4.9	8.3
Milk yield^4^^,^ ^10^	9,047	4,362–11,622	8,238	9,170	9,974	7,538	3,940–10,482	6,893	7,600	8,338
Milk fat^4^^,^ ^10^	370.0	202.0–457.0	345.0	375.0	404.0	312.4	161.0–434.6	284.8	316.2	343.6
Milk protein^4^^,^ ^10^	307.0	145.0–392.0	283.0	315.0	339.0	263.6	128.4–370.4	240.6	267.4	294.3
SCC^4^^,^ ^11^	219.0	123.0–664.0	187.0	212.0	241.0	205.0	106.2–421.8	166.6	197.6	230.9
Calving interval^12^	414.0	359.0–552.0	399.0	409.0	422.0	396.0	355.8–471.8	376.5	392.1	411.5
Herd size	95	10–486	51	79	115	46.5	5.0–231.0	27.0	40.5	59.0

^1^ Farm level prevalence of underconditioned cows in %

^2^ Farm level prevalence of optimally conditioned cows in %

^3^ Farm level prevalence of overconditioned cows in %

^4^ Bayesian bootstrap

^5^ Farm level prevalence of contaminated udders in %

^6^ Farm level prevalence of contaminated legs in %

^7^ Farm level prevalence of hock lesions (swellings and/or wounds) in %

^8^ Farm level prevalence in %

^9^ farm level prevalence of amputated tails and tails with deviation and/or swelling/bulge

^10^ in kg

^11^ × 1000 cells/ml

^12^ in days

**Table 2:** Descriptive statistics of categorical variables within the imputed data set for each of the two study regions (North = 188 farms, South = 212 farms).

Variable	Categories	North		South	
		Counts (n_farms_)	Percent (%_farms_)	Counts (n_farms_)	Percent (%_farms_)
Pasture access	No	38	20.2	140	66.0
	Yes	150	79.8	72	34.0
Exercise area	No	136	72.3	162	76.4
	Yes	52	27.7	50	23.6
Housing	Tie stall	-	-	54.	25.5
	Free stall	161	86.6	151.	71.2
	Pasture based system	14	7.5	-	-
	Other	13	6.9	7	3.3
Farming type	Conventional	182	96.7	179	84.4
	Organic	6	3.2	33	15.6
Main/Sidelinea^1^	Main	186	98.9	179	84.4
	Sideline	2	1.1	33	15.6
Study year	1	64	34.0	83	39.2
	2	74	39.4	84	39.6
	3	50	26.6	45	21.2
Satisfaction animal health^2^	Disagreement	34	18.1	27	12.7
	Neutrality	36	19.1	40	18.9
	Agreement	118	62.8	145	68.4
Strain^3^	Disagreement	106	56.4	107	50.5
	Neutrality	37	19.7	51	24.1
	Agreement	45	23.9	54	25.5
Emotional relationship^4^	Disagreement	16	8.5	22	10.4
	Neutrality	25	13.3	22	10.4
	Agreement	145	78.2	168	79.2
Continuing education^5^	Disagreement	24	12.8	19	9.0
	Neutrality	16	8.5	27	12.7
	Agreement	148	78.7	166	78.3
Facial expression^6^	In all/most cases	28	14.9	84	39.6
	In suspicious cases	118	62.8	86	40.6
	Rarely/never	42	22.3	42	19.8
Temperature^7^	In all/most cases	53	28.2	83	39.2
	In suspicious cases	25	13.3	17	8.0
	Rarely/never	110	58.5	112	52.8
Udder control^8^	In all/most cases	14	7.5	-	-
	In suspicious cases	157	83.5	-	-
	Rarely/never	17	9.0	-	-
Vitamins^9^	In all/most cases	142	75.5	-	-
	In suspicious cases	20	10.6	-	-
	Rarely/never	26	13.8	-	-
HHS^10^	No	92	48.9	169	79.7
	Yes	96	51.1	43	20.3
Documentation^11^	No	144	76.6	177	83.5
	Yes	44	23.4	35	16.5

^1^ Dairy farming as main source of income or sideline/supplementary source of income

^2^ “I am satisfied with the animal health situation on my farm”

^3^ “My daily work puts strain on me”

^4^ “I can imagine myself building an emotional relationship with a cow”

^5^ “I regularly attend events and conferences of continuing education”

^6^ “I have a look at the facial expression and the eyes of my cows during the period around calving”

^7^ “I check body temperature using a thermometer”

^8^ “I check the udder after calving”

^9^ “I preventively administer vitamins and minerals”

^10^ Herd Health Services; “I am enroled to systematic and professional herd health services”

^11^ “I document cases of health issues in a written form”

BTM antibody data were available for 200 farms. After removal of 12 farms which were not enrolled to DHI, the final data set for analysis comprised of a total of 17,806 dairy cows on 188 farms housing a mean of 95 cows (range 10.0–486.0; median 79.00). Missing values were imputed for scorings, attitude variables, and management measures around calving. Altogether, 161 farms (85.6%) were free-stall operations, 14 pasture-based systems (7.5%), and 13 farms (6.9%) were assigned to the “other” category containing deep straw-bedded packs and tied housing. Organic farming principles were adhered to on six farms (3.2%) and for two farms(1.1%) dairy farming represented a sideline income which was the reason to exclude these variables from the data set due to the low number of observations. Cows had access to pasture on 150 farms (79.8%) and to an outdoor exercise area on 52 farms (27.7%). *Fasciola hepatica* antibodies were confirmed in BTM samples of 28 farms (15.0%). As for the attitude data, the variables Animal handling easy, *Care male calves*, *Patience*, *Discussion improvements*, and *Pain* were excluded due to only few observations in some categories. Likewise the variables *BHB check* and *BCS check* were excluded. Tables 3 and 4 display the descriptive results of the complete cases data set.

**Table 3:** Descriptive statistics of continuous variables within the complete cases data set for each of the two study regions (North = 179 farms, South = 207 farms).

	North					South				
Variable	Mean	Range	1^st^ Qu.	Median	3^rd^ Qu.	Mean	Range	1^st^ Qu.	Median	3^rd^ Qu.
Underconditioned^1^	20.6	0.0–100.0	12.5	19.8	27.8	10.0	0.0–61.8	4.0	8.3	14.3
Optimally conditioned^2^	68.9	0.0–91.9	64.7	69.8	75.2	71.5	35.3–100.0	62.6	71.7	80.9
Overconditioned^3^	10.5	0.0–100.0	3.6	7.5	13.8	17.7	0.0–60.7	7.8	15.7	25.0
Parity^4^	2.5	1.6–3.7	2.3	2.4	2.6	2.5	1.7–3.7	2.3	2.5	2.7
Udder hygiene^5^	22.0	0.0–85.7	9.9	19.4	30.1	21.4	0.0–76.3	9.7	19.2	21.4
Leg hygiene^6^	40.4	0.0–95.2	22.2	34.8	57.9	32.3	0.0–88.5	18.6	29.2	43.3
Hock lesions^7^	13.7	0.0–47.5	4.7	11.1	20.7	15.7	0.0–77.8	6.0	12.5	23.9
Lameness^8^	26.0	0.0–76.9	15.1	23.0	26.0	24.5	0.0–67.6	14.4	23.1	33.0
Tail changes^9^	12.3	0.0–53.3	6.8	9.9	15.6	5.8	0.0–28.9	2.0	4.8	8.1
Milk yield^4, 10^	9,079	4,362–11,622	8,307	9,174	9,954	7,552	3,940–10,482	6,914	7,611	8,350
Milk fat^4^^,^ ^10^	370.3	202.1–458.5	347.1	374.5	402.8	312.7	161.0–434.6	285.8	316.4	343.7
Milk protein^4^^,^ ^10^	308.1	145.2–392.0	283.7	315.1	308.1	264.1	128.4–370.4	241.2	267.8	295.0
SCC^4^^,^ ^11^	219.4	122.9–663.9	184.8	211.8	242.4	203.3	106.2–421.8	166.1	195.6	225.5
Calving interval^12^	412.6	359.3–552.4	398.7	407.9	420.0	395.8	355.8–471.8	376.3	392.1	411.4
Herd size	97.3	17.0–486.0	57.5	84.0	116.0	46.2	5.0–231.0	27.0	40.0	58.5

^1^ Farm level prevalence of underconditioned cows in %

^2^ Farm level prevalence of optimally conditioned cows in %

^3^ Farm level prevalence of overconditioned cows in %

^4^ Bayesian bootstrap

^5^ Farm level prevalence of contaminated udders in %

^6^ Farm level prevalence of contaminated legs in %

^7^ Farm level prevalence of hock lesions (swellings and/or wounds) in %

^8^ Farm level prevalence in %

^9^ farm level prevalence of amputated tails and tails with deviation and/or swelling/bulge

^10^ in kg

^11^ × 1000 cells/ml

^12^ in days

**Table 4:** Descriptive statistics of categorical variables within the imputed data set for each of the two study regions (North = 179 farms, South = 207 farms).

Variable	Categories	North		South	
		Counts (n_farms_)	Percent (%_farms_)	Counts (n_farms_)	Percent (%_farms_)
Pasture access	No	40	22.3	136	65.7
	Yes	139	77.3	71	34.3
Exercise area	No	133	74.3	157	75.8
	Yes	46	25.7	50	24.1
Housing	Tie stall	-	-	53	25.6
	Free stall	155	86.6	147	71.0
	Pasture based system	12	6.7	-	-
	Other	12	6.7	7	3.4
Farming type	Conventional			173	84.1
	Organic			33	15.9
Main/Sidelinea^1^	Main	-	-	174	84.1
	Sideline	-	-	33	15.9
Study year	1	61	34.1	80	38.6
	2	71	39.7	82	39.6
	3	47	26.3	45	21.7
Satisfaction animal health^2^	Disagreement	30	16.8	26	12.6
	Neutrality	33	18.4	38	18.4
	Agreement	116	64.8	143	25.1
Strain^3^	Disagreement	94	52.5	104	50.2
	Neutrality	41	22.9	51	24.6
	Agreement	44	24.6	52	25.1
Emotional relationship^4^	Disagreement	16	8.9	22	10.6
	Neutrality	28	15.6	21	10.1
	Agreement	135	75.4	164	79.2
Continuing education^5^	Disagreement	22	12.3	19	9.2
	Neutrality	17	9.5	25	12.1
	Agreement	140	78.2	163	78.7
Facial expression^6^	In all/most cases	29	16.2	83	40.1
	In suspicious cases	109	60.9	84	40.6
	Rarely/never	41	22.9	40	19.3
Temperature^7^	In all/most cases	52	29.1	82	39.6
	In suspicious cases	19	10.6	17	8.2
	Rarely/never	108	60.3	108	52.2
Udder control^8^	In all/most cases	11	6.1	-	-
	In suspicious cases	150	83.8	-	-
	Rarely/never	18	10.1	-	-
Vitamins^9^	In all/most cases	133	74.3	-	-
	In suspicious cases	19	10.6	-	-
	Rarely/never	27	15.1	-	-
HHS^10^	No	87	48.6	165	79.7
	Yes	92	51.4	42	20.3
Documentation^11^	No	135	75.4	173	83.6
	Yes	44	24.6	34	16.4

^1^ Dairy farming as main source of income or sideline/supplementary source of income

^2^ “I am satisfied with the animal health situation on my farm”

^3^ “My daily work puts strain on me”

^4^ “I can imagine myself building an emotional relationship with a cow”

^5^ “I regularly attend events and conferences of continuing education”

^6^ “I have a look at the facial expression and the eyes of my cows during the period around calving”

^7^ “I check body temperature using a thermometer”

^8^ “I check the udder after calving”

^9^ “I preventively administer vitamins and minerals”

^10^ Herd Health Services; “I am enroled to systematic and professional herd health services”

^11^ “I document cases of health issues in a written form”

The complete cases data set comprised of 179 farms housing 17,410 cows with a mean of 97 cows per farm (range 17.0–486.0; median 84.0). One hundred and fifty-five farms (86.6%) had free stall housing compared with twelve pasture-based operations (6.7%), and twelve farms (6.7%) in the “other” category. Pasture access was granted on 139 farms (77.7%) and an outdoor exercise was present on 46 farms (25.7%). *Fasciola hepatica* antibodies were detected on 29 farms (16.2%).

A total number of 1,000 trees were grown in the RF with three variables being the best number of variables to be tried at each split. All features incorporated in the RF are provided in Table 5. The OOB was 14.0%. Precision of the RF was 98.3% and predictive accuracy 86.5%. Recall and F1 score appeared to be 86.5% and 92.8%, respectively.

**Table 5:** Compilation and description of all features included in the random forest models.

Feature	Feature type	Explanation
Calving Interval	Continuous	Farm level median of calving interval
Continuing education	Categorical	Farmer confirms regular participation in events and/or conferences of continuing education; categories: disagreement, neutrality, agreement
Documentation	Categorical	Documentation of cases of health issues in a written form by farmer; yes/no
Emotional relationship	Categorical	Farmer can imagine building an emotional relationship with a cow; categories: disagreement, neutrality, agreement
Exercise	Categorical	Presence of an outdoor exercise area for cows; yes/no
Farming type	Categorical	Conventional vs. organic farming
Facial expression	Categorical	Farmer has a look at the facial expression and the eyes of cows during the period around calving; categories: in all/most cases, in suspicious cases, rarely/never
Herd size	Continuous	Number of lactating and dry cows
HHS	Categorical	The farm is enrolled to systematic and professional herd health servises; yes/no
Hocks	Continuous	Farm level prevalence of hock lesions (more severe lesions, i.e. swelling and/or wound)
Housing	Categorical	Housing type;
		region North: Free stall housing, pasture-based systems, other (tied housing, straw-bedded packs)
		region Soutch: Free stall housing, tie stall housing, other (e.g. straw-bedded packs)
Lameness	Continuous	Farm-level lameness prevalence based on locomotion score ≥ 3 (loose housing) and Stall Lameness Score ≥ 2 (tied housing), respectively
Leg hygiene	Continuous	Farm-level prevalence of contaminated lower legs (considerable contamination, score ≥ 3)
Main sideline	Categorical	Dairy farming as main or sideline source of income
Milk fat	Continuous	Farm-level median of milk fat
Milk protein	Continuous	Farm-level median of milk protein
Milk yield	Continuous	Farm-level median of milk yield
Optimally conditioned	Continuous	Farm-level prevalence of optimally conditioned cows
Overconditioned	Continuous	Farm-level prevalence of overconditioned cows
Parity	Continuous	Farm-level median of parity
Pasture	Categorical	Presence of pasture access; yes/no
Satisfaction animal health	Category	Farmer expresses satisfaction with the animal health situation on the farm; categories: disagreement, neutrality, agreement
SCC	Continuous	Farm-level median somatic cell count
Season	Categorical	Spring, summer, fall, winter
Strain	Categorical	Farmer expresses that the daily farm wotk puts strain on them; categories: disagreement, neutrality, agreement
Tail	Continuous	Farm-level prevalence of tail lesions (visible changes, i.e. deviance of axis and/or bulge/swelling, amputation)
Temperature	Category	Farmer checks body temperature of cows during the period around calving using a thermometer; categories: in all/most cases, in suspicious cases, rarely/never
Udder control	Categorical	Farmer checkst he udder of cows after calving; categories: in all/most cases, in suspicious cases, rarely/never
Udder hygiene	Continuous	Farm-level prevalence of contaminated udders (considerable contamination ≥ score 3)
Underconditioned	Continuous	Farm-level prevalence of underconditioned cows
Vitamins	Categorical	Farmer preventivela administers vitamins and minerals to cows durin the period around calving; categories: in all/most cases, in suspicious cases, rarely/never
Year	Categorical	Study years 1, 2, and 3

**Fig 1:**
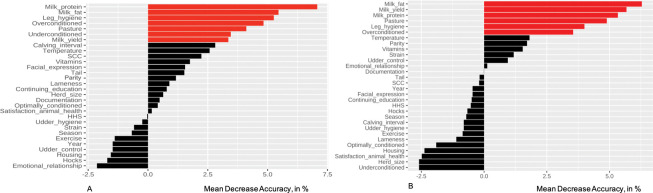
Variable importance plot for the random forest models in study region North. Predictors with a permutation p-value ≤ 0.05 are highlighted in red. The higher the Mean Decrease Accuracy value (on the x-axis, in %), the more the predictive accuracy of the model would suffer if removing the respective predictor. In cases of negative permutation importance values, permutation revealed that accuracy of the permutated data was superior to the real data which translates into the irrelevance of the respective variables. A: Results for the RF model on the imputed data (n_farms_ = 188); B: Results of the RF model on the complete cases data set (n_farms_ = 179).

Fig 1A displays the ranked variables of the random forest of the imputed data with seven factors (p ≤ 0.05) relevant for on-farm *F*. *hepatica* seropositivity highlighted in red.

Next to the top ranking variable farm-level milk protein (MDA 7.1%, p = 0.002), farm-level milk fat (MDA 5.5%, p = 0.006), leg hygiene (MDA 5.3%, p = 0.02), and prevalence of overconditioned cows (MDA 4.8%, p = 0.02) appeared among the top-ranking covariates. Further relevant features were pasture (MDA 4.1%, p = 0.02), prevalence of underconditioned cows (MDA 3.5%, p = 0.04), and farm-level milk yield (MDA 3.4%, p = 0.02). The RF results for the complete cases data set are illustrated in Fig 1B. The forest was grown with a precision of 97.9%, a predictive accuracy of 85.2%, a recall of 86.6%, and a F1 score of 92.0%. Production trait related factors represented the top three important features starting with farm-level milk fat (MDA 6.3%, p = 0.001), followed by farm-level milk yield (MDA 5.7%, p = 0.004), and farm-level milk protein (MDA 5.3%, p = 0.005). Pasture (MDA 4.9%, p = 0.007) was the third top-ranking feature. Similarly to the model on the imputed data, leg hygiene (MDA 4.0, p = 0.04) and prevalence of overconditioned cows (MDA 3.5%, p = 0.03) appeared among the most important features.

### Region South

Descriptive results of the imputed data set are illustrated in Tables 1 and 2. Parasitological data were obtained from 240 out of 260 farms. Since 28 farms did not participate in DHI, they were removed and the data set for analysis consisted of 212 farms housing 9,847 dairy cows with a mean of 46 cows (range 5.0–231.0, median 41.0). A total number of 54 farms (25.5%) housed their cows in tie stalls, whereas 151 farms (71.2%) were free-stall operations and seven farms (3.3%) were assigned to the “other” category. Organic farming was present on 33 farms (15.6%) and for 33 farms (15.6%) dairy production represented a sideline source of income. Cows had pasture access on 72 farms (34.0%) and an outdoor exercise area on 50 farms (23.6%). Fifty farms (23.6%) were positive for *F*. *hepatica* in BTM samples.

The complete cases data set comprised of 207 farms housing 9,570 cows with a mean herd size of 46 cows (range 5–231, median 40.0). Descriptive statistics are compiled in Tables 3 and 4. Free stall housing was provided on 147 farms (71.05), 53 farms (25.6%) tied their cows, and seven farms (3.4%) were in the “other” category. Thirty-three of the farms were organic (15.9%) and 174 farms (84.1%) adhered to conventional farming practices. On 33 farms (15.9%), dairy farming was a sideline income, whereas 174 farms (84.1%) relied on dairy farming as the only source of income. Pasturing grounds were provided to cows on 71 farms (34.3%) and an outdoors exercise area was present on 50 operations. (24.2%). *Fasciola hepatica* was present on 49 farms (23.7%).

**Fig 2:**
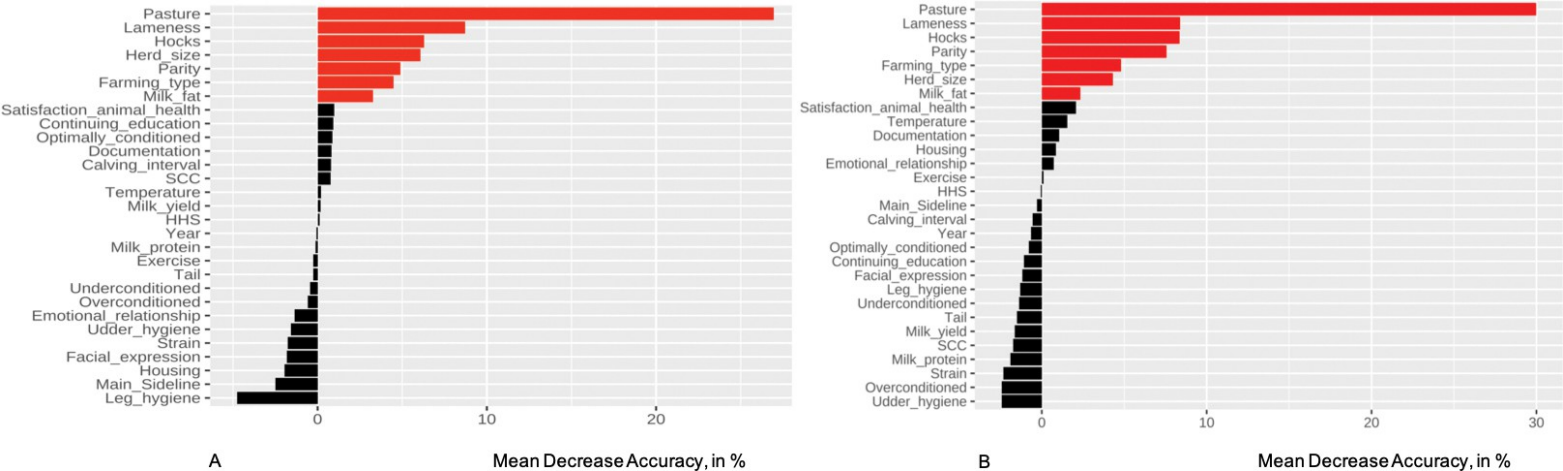
Random forest model results for study region South. Predictors with a permutation p-value ≤ 0.05 are highlighted in red. The higher the Mean Decrease Accuracy value (on the x-axis, in %), the more the predictive accuracy of the model would suffer if removing the respective predictor. In case of negative permutation importance values, permutation revealed that accuracy of the permutated data was superior to the real data which translates into the irrelevance of the respective variables for the assessed setting. A: Results for the RF model on the imputed data (n_farms_ = 212); B: Results of the RF model on the complete cases data set (n_farms_ = 207).

Similar to Region North, the variables *Animal handling easy*, *Care male calves*, *Patience*, *Discussion improvements*, *Pain*, *BHB check*, and *BCS check* were excluded due to only few observations in some categories. Additionally, *Udder control* and *Vitamins* were excluded for the same reason. Results of the RF are presented in Fig 2 with relevant (p ≤ 0.05) factors highlighted in red.

The RF was grown with 1,000 trees and four variables appeared to be optimal to be tried at each split yielding an OOB error of 13.7%. The forest of the imputed data was generated with a precision of 77.8%, a predictive accuracy of 83.1%, a recall of 100%, and a F1 score of 87.5%. With a MDA of 30.0% (p = 0.001), pasture turned out to be by far the most important factor to describe the presence of *F*. *hepatica* on farm level. Farm-level lameness prevalence was the second highest ranking variable (MDA 8.7%, p = 0.005 followed by farm-level prevalence of hock lesions (MDA 6.3%, p = 0.008). Other relevant features were herd size (MDA 6.1%, p = 0.005), parity (MDA 4.9%, p = 0.03), farming type (MDA 4.5%, p = 0.007), and farm-level milk fat (MDA 3.3%, p = 0.009). As for the complete cases data set, the model performance covered a precision of 98.0%, a predictive accuracy of 87.3%, a recall of 87.3%, and a F1 score of 92.3%. Pasture was the top ranking variable with a MDA of 30.0% (p = 0.001) followed by farm-level lameness prevalence (MDA 8.4%, p = 0.003), farm-level prevalence of hock lesions (MDA 8.4%, p = 0.002), parity (MDA 7.6%, p = 0.008), farming type (MDA 4.8%, p = 0.004), herd size (MDA 4.3%, p = 0.02), and milk fat (MDA 2.3%, p = 0.02).

## Discussion

*Fasciola hepatica* has a complex lifecycle and a volatile epidemiology which render prediction on farm level challenging. Forecasting models to assess the risk of individual farms have focused on seasonal and climate-driven factors [14, 54, 55]. The problem encountered by all of these models is the limited applicability to other regions, their restricted ability to extrapolate conclusions, and their insufficient validity on farm level [54]. In the absence of holistic prediction models taking into account the interplay of different factors associated with parasite occurrence, optimal, farm-specific control strategies cannot be identified and additional efforts are necessary to address the complex epidemiology of fasciolosis and to identify key parameters [22]. Bennema et al. [21] have drawn attention to farm-specific management factors, which may play a role in regard to predicting infection risk. Using two region-specific data sets covering a total number of 27,653 dairy cows on 400 farms across Germany as well as including comprehensive information on production parameters, husbandry methods, management regimes, and farmer attitude, we were able to apply a RF approach and to identify and rank relevant features related to parasite occurrence on farm level.

A substantial characteristic of RF algorithms is the provision of variable importance measures illustrating the degree of association between a certain covariate and the response. This allows for the evaluation of a set of available features and their differential importance in regard to the target. Hence, in an epidemiological situation such as fasciolosis with a life cycle involving several stages subjected to a plethora of features associated with parasite development, intermediate host occurrence, and transmission between host species [20, 23, 26], RF allows to determine those factors that are most important for the presence of *F*. *hepatica* in the first place. Random forest algorithms are robust to outliers, noise, and overfitting and make no assumptions about the data being analysed [26, 56]. Moreover, a factor can be important to the system based on its relevance also to other input variables and hence be high ranking without being statistically significant and detached from the idea of the direction of the association between predictor and target. This allows for a holistic view of an epidemiological situation considering its complexity and the interplay among variables. Therefore, RF appeared to be an intuitive approach to model the occurrence of *F*. *hepatica* based on farm characteristics and to identify key factors associated with farm-level presence of the parasite. Given the situation that data from two structurally different dairy regions were analysed in this study, the results have the advantage that they can be extrapolated to similar settings of dairy production acknowledging the respective region-specific characteristics and aspects that merit consideration.

In the context of data collection of the present study, a comprehensive set of covariates was assessed and information in regard to these factors was recorded. At first glance, a number of factors included in the RF models might not intuitively appear to be relevant for the presence of *F*. *hepatica*. Yet, when conducting the presented analyses, we did not intend to restrict the inclusion of covariates on an a-priori judgement of their relevance, but we rather aimed at including as many of these features as possible and specifically also those that might not have been known to be associated with *F*. *hepatica*. As the results of this study confirm, a number of factors indeed turned out to not be important for the outcome and during the analyses removal or addition of these factors did not yield an improvement nor a deterioration of model performance. Based on the results of the present work, hence the next step would be a specific study with a comprehensive set of features with relevance to the biology and epidemiology of *F*. *hepatica* incorporating the aspects identified as important in the course of the present work.

In the model of the imputed data for southern herds, recall was 100%. A perfect recall means that false negatives are not existent in the model classification and every negative prediction thus is correct. Given the data where the number of positives, i.e. farms seropositive for *F*. *hepatica*, is relatively low in relation to the negatives, i.e. farms seronegative for *F*. *hepatica*, correct classifications and an optimised model performance were crucial. We therefore applied several steps to optimise model performance and obtain the highest possible model performance parameters. On the data level, the data were elaborately checked for plausibility and cleaned prior to the analyses and subsequently edited to enter the modelling procedure. As another element of model optimisation, different splitting ratios of the data were examined in order to determine the one ratio that would yield the best model performance. Model performance parameters then were tuned using a manual approach. Different values for the number of trees to grow, for the number of variables to be randomly sampled as candidates at each split, and for the number of permutation replicates to run were evaluated also in different combinations in order to obtain the best possible model performance parameters. The manual approach was particularly feasible in the present work, since the number of parameters to be defined was relatively small. Yet, it is very important to be aware fo the fact that this opens the possibility to introduce human bias since potential combinations of parameters could have been overlooked. Given the very satisfactory outcome of the models and the fact that extensive time and effort was dedicated to optimise model performance at several levels, this bias yet may well have been minor.

Pasture access appeared to be among the most important features in all models across both regions. Considering the epidemiology of *F*. *hepatica* which relies on the ingestion of infective metacercariae via vegetation to complete its life cycle [57], this finding is little surprising. Nevertheless, pasture access represents a certain type of dairy farming in region South which may hence mediate its relevance in regard to *F*. *hepatica*. For instance, farms offering pasture access may differ considerably in their management practices and housing facilities from operations on which pasturing is not performed. Furthermore, pasture access may be the prerequisite for other identified factors to come into play when occurring together and creating a certain on-farm setting. This thought is further corroborated by two other high ranking variables of importance, i.e. farming type and herd size. Herd size has previously been associated with the presence of *F*. *hepatica* [7]. Additionally, herd size in region South probably is a proxy for farm characteristics meaning that large farms may be more intensely managed with a greater level of industrialisation whereas small farms are more likely to be run according to organic principles and may more frequently incorporate pasture access. Accordingly, these characteristics may translate into the risk of *F*. *hepatica* presence on the farm being more distinct on organic operations.

Farm-level lameness prevalence was the second highest ranking variable describing *F*. *hepatica* presence in region South. Lameness is a widespread issue in dairy production and has negative implications for animal health, welfare, and productivity [58, 59]. Lameness has furthermore been associated with housing conditions and farm management [60, 61]. In particular, organic farming (i.e. farming type) and pasture access, both top-ranking variables in study region South, have been demonstrated to be beneficial for lameness dynamics within a herd [62]. Likewise, pasture access has been identified to lower the odds of dirty legs [63]. Leg hygiene ranked third in the imputed data model and fifth in the complete data model in study region North, not in the study region South, though. Nevertheless, it is not surprising that pasture access, which lowers the risk for lameness and for soiled legs [31, 63, 64], increases the risk for dairy herds to be infected with *F*. *hepatica*. The result of hock lesions ranking third in study region North complements the reasoning elaborated on above. Hock lesions are common in dairy cows and have been associated with lameness due to common risk settings favoring their occurrence [31].

Production parameters, i.e. farm-level milk yield, milk fat, and milk protein appeared among the top ranking features linked to the presence of *F*. *hepatica* in both the imputed and the complete cases data as well as in both study regions. Previous studies have elucidated associations between production parameters and the presence of *F*. *hepatica* [65, 66]. According to Mezo et al. [67], a mean of 1.5 kg of milk loss is to be expected in cows per day when a strong infection level is present on farm. Moreover, adverse effects on milk fat and milk protein have been associated with the parasite [68].

Median farm level parity was a relevant predictor in study region South. Since the parasite can persist for more than two years within the host [11, 69], depending on the time point of infection, positive results can stem from cows in a higher parity being persistently infected or reinfected. The idea of higher age being associated with seropositivity is supported by Pinilla et al. [70] who have identified a higher risk of infection in animals older than one year compared with younger cattle. Moreover, seropositivity may mainly be traced back to younger cows in lower parities which remain seropositive from getting infected as heifers. This may be particularly the case in study region South, since it is common to rear youngstock on more remote pastures and alpine pastures, which usually are exposed to geographical and meteorological risk factors for fasciolosis different from those present on their home farm. Hence, the relevance of median parity level in the present study could be mediated by heifers and young cows carrying infections. This finding specific to region South explicitly demonstrates that infection sources differ between both studied regions, as the environmental differences young stock and adult cows are exposed to are less profound in region North. Additionally, the age-dependent theory outlined above could also be applied to region North where yet parity appeared not to be a top ranking feature.

Body Condition Score appeared among the relevant features in the models in study region North. Interestingly, both overcondition and undercondition made it to the top in northern herds (imputed data) and only overcondition appeared in the complete cases data model which seems contradictory in the first place. Yet, since body condition has frequently been used as an indicator of animal health, and previous studies have acknowledged the association of *F*. *hepatica* with body condition in cattle [65, 71] supporting the outcome of the present RF models, this result emphasises the importance of this feature. Probably, any body condition of cows that is below or above the optimum may either predisope these animals to disease or be an indicator for the presence of disease. Moreover, in the context of the present work, suboptimal body condition may also act as a proxy for other husbandry-associated characteristics that may translate into relevance for *F*. *hepatica* presence as well.

In order to correctly interpret our results, some limitations to this study are to be considered. Firstly, a cross-sectional study design was pursued throughout data collection which entails some inherent limitations [72, 73]. Since predictors and outcomes are recorded at the same time, observer bias might enter the data collection process. We addressed this issue by following strict and rigorous standard operating procedures throughout the study period which were continuously reassessed during the data collection period in order to ensure uniform and unbiased data acquisition. Furthermore, a three-month pilot phase prior to the actual start of data collection was launched in order to identify potentially challenging points in the interview questionnaires or data entry forms and to subsequently modify procedures accordingly. Thirdly, weekly telephone conferences among the study teams were conducted in order to discuss upcoming topics and to identify challenges during data acquisition. Given these measures, we are convinced that the introduction of bias through observers can be considered minor. As a second point, a cross-sectional study design does not allow to draw inference on causal relationships between covariates and outcomes. Specific study designs are required to further dwell into the causalities within the data set. Thirdly, the voluntary participation of farmers and the required proactivity to get in touch with the study team may have created a certain level of selection bias. Proactive, open minded farmers who are interested in recent scientific findings and who are willing to constantly improve animal welfare and husbandry on their operations based on the state-of-the-art in the field may have been more encouraged to participate which may subsequently have translated into overall improved animal health situations compared with the true underlying population. On the other hand, farmers who were confronted with health issues in their herds and hence saw an opportunity for external consultation may have been more inclined to participate. As a consequence, the study population could yield higher prevalences of disease than actually present in the target population. A certain level of selection bias might be the reason why not enough variation was seen in a considerable part of the attitude and management-related variables which eventually led to the exclusion of these factors due to low numbers of observations in certain categories. Secondly, this bias could have entailed that the majority of factors related to farmer attitude and on farm management procedures have not appeared to be relevant even though previous studies have determined farmer attitude to be a crucial part in decision-making processes and can be related to animal health. Although we cannot exclude some extent of selection bias in this context, we still believe it has been considerably reduced by the sampling procedure.

Using questionnaires and entry forms for data collection has been shown to entail missing or inconsistent data [74] which impairs unbiased data analysis and negatively interferes with machine learning algorithms [75]. Removing incomplete rows from the data set is associated with considerable loss of information and may lead to skewed results and decreased statistical power of the analysis. Multiple imputation approaches allow for the estimation of parameters assuming a missing at random mechanism present in the data. This means that the mechanism of missingness can solely be traced back to the data. On the other hand, data may not be missing at random but be missing due to unobserved aspects. However, it is virtually impossible to determine the true missingness mechanism present in the data [76, 77]. In the present study, missing values for single variables were present both at cow and herd level. If the percentage of missing values for a single variable did not exceed a threshold of 10%, we conducted a non-parametric, multivariate imputation via chained random forest in order to address this issue. Missing values were not imputed for the target. This allowed for the inclusion of a larger number of farms which otherwise would have been lost for analysis and increased reliability, power, and transferability of the analysis. Yet, this approach could be associated with potential inaccuracy and necessitates further steps to ensure reliable inference from the analyses. Therefore, we conducted each anaylsis in a duplicate setting. After identification of the optimal model parameters, one model per study region was fitted using the imputed data set and one using the complete cases data set without imputations. This enabled us to compare the outcomes of both models, to identify potential congruences or disagreements between models which eventually strengthensa the reliability of the results. According to Héraud-Bousquet et al. [76], sensitivity analyses for the exploration of the robustness of inference are important in cases where missing data are present and imputations are conducted. In the current work, the variables with the highest percentages of missing values were imputed at cow level and subsequently the information was raised to farm level, i.e. farm level prevalences, which probably were not abundantly susceptible to an outstanding level of bias induced by the imputed values additionally because the missing values at cow level were also distributed across farms. Moreover, for the variables imputed at farm level, the percentage of missing values that were imputed were less than 2% of the data. This was the reason why we chose not to conduct a sensitivity analysis as suggested and rather compared the model results of the RF of the imputed data with the results of a model fitted on a complete cases data set. Both the models of the imputed data as well as the models of the complete cases data largely agreed on the importance of the top ranking features which clearly emphasises the relevance of these factors. Some factors that appeared in the imputed data models but not in the ones on the complete data might be spurious and their relevance needs to be regarded with caution despite seeming plausible. Here, some inaccuracy might have entered the analysis, but this may be traced back both to potential bias through the imputations as wells to potential bias in the complete cases data set. Therefore, it needs to be regarded as a limitation that a sensitivity analysis was not reproduced as outlined by Héraud-Bousquet et al. [76]. As the win of accuracy, reliability, and possibility of extrapolation clearly outweighs the potential drawbacks of the imputation and given the congruence of the models in the ranking of the centrally important features, we are confident that the model results presented here are valid and robust.

Faecal analysis as well as individual milk samples would have increased the knowledge gain and would have allowed to generate a more holistic view of the parasitological situation on a given farm. Furthermore, these data would have allowed to include site-specific meteorlogical data into the analysis. Yet, this was beyond the scope of data collection in the context of this study and could not be realised within the frame of the present work. Therefore, this may well be considered in future work.

## Supporting information

S1 Table Overview of missing values per variable within study region for all imputed features within the models. (DOCX)

S2 Table Attitude and management-related questions and respective variables. (DOCX)

S3 Table List of R packages. All implemented R packages including references.
(DOCX)

S1 Data Complete cases data set of study region North. (CSV)

S2 Data Complete cases data set of study region South. (CSV)
