# Supplementary material for: Random forest classification as a tool in epidemiological modelling: Identification of farm-specific characteristics relevant for the occurrence of Fasciola hepatica on German dairy farms
Source: PLoS One. 2023 Dec 21;18(12):e0296093. doi: 10.1371/journal.pone.0296093 (PMC10735020; doi:10.1371/journal.pone.0296093)
Supplement: S1 Table — (DOCX) [file pone.0296093.s001.docx]

**S1 Table. Overview of missing values per variable within study region for all imputed features within the models.**

| Study region North | | | |
| --- | --- | --- | --- |
| Variable | Level | Number of missing values (n) | Percentage (%) |
| Tail | Individual cow | 109 | 0.44 |
| Udder hygiene | Individual cow | 180 | 0.72 |
| Leg hygiene | Individual cow | 493 | 1.97 |
| Hocks | Individual cow | 1,567 | 6.27 |
| Emotional relationship | Farm | 2 | 1.06 |
| Continuing education | Farm | 2 | 1.06 |
| Satisfaction animal health | Farm | 2 | 1.06 |
| Strain | Farm | 4 | 2.13 |
| Vitamins | Farm | 3 | 1.6 |
| Study region South | | | |
| Tail | Individual cow | 11 | 0.1 |
| Udder hygiene | Individual cow | 15 | 0.13 |
| Leg hygiene | Individual cow | 26 | 0.23 |
| Hocks | Individual cow | 1,126 | 9.85 |
| Emotional relationship | Farm | 1 | 0.47 |
| HHS | Farm | 1 | 0.47 |
| Facial expression | Farm | 1 | 0.47 |
