# Supplementary material for: Random forest classification as a tool in epidemiological modelling: Identification of farm-specific characteristics relevant for the occurrence of Fasciola hepatica on German dairy farms
Source: PLoS One. 2023 Dec 21;18(12):e0296093. doi: 10.1371/journal.pone.0296093 (PMC10735020; doi:10.1371/journal.pone.0296093)
Supplement: S2 Table — (DOCX) [file pone.0296093.s002.docx]

**S1 Table. Attitude and management related questions and respective variables.**

| Attitude | | |
| --- | --- | --- |
| Statement | Potential Rating by farmers | Variable denomination |
| “I am satisfied with the animal health situation on my farm” | 0 = strongly disagree  1 = disagree  2 = neutral  3 = agree  4 = strongly agree | Satisfaction animal health |
| “My daily work puts strain on me” | 0 = strongly disagree  1 = disagree  2 = neutral  3 = agree  4 = strongly agree | Strain |
| “Handling the animals is easy for me” | 0 = strongly disagree  1 = disagree  2 = neutral  3 = agree  4 = strongly agree | Animal handling easy |
| “I can imagine myself building an emotional relationship with a cow” | 0 = strongly disagree  1 = disagree  2 = neutral  3 = agree  4 = strongly agree | Emotional relationship |
| “I exert the same care for male calves as I exert for female offspring” | 0 = strongly disagree  1 = disagree  2 = neutral  3 = agree  4 = strongly agree | Care male calves |
| “I regularly attend events and conferences of continuing education” | 0 = strongly disagree  1 = disagree  2 = neutral  3 = agree  4 = strongly agree | Continuing education |
| “It is important to me to be patient with the animals | 0 = strongly disagree  1 = disagree  2 = neutral  3 = agree  4 = strongly agree | Patience |
| “On our farm, we regularly have critical discussions about potential improvements” | 0 = strongly disagree  1 = disagree  2 = neutral  3 = agree  4 = strongly agree | Discussion improvements |
| “It affects me to see pain in the animals” | 0 = strongly disagree  1 = disagree  2 = neutral  3 = agree  4 = strongly agree | Pain |
