# Supplementary material for: Random forest classification as a tool in epidemiological modelling: Identification of farm-specific characteristics relevant for the occurrence of Fasciola hepatica on German dairy farms
Source: PLoS One. 2023 Dec 21;18(12):e0296093. doi: 10.1371/journal.pone.0296093 (PMC10735020; doi:10.1371/journal.pone.0296093)
Supplement: S3 Table — All implemented R packages including references. (DOCX) [file pone.0296093.s003.docx]

**S2 Table**. List of R packages, alphabetical order.

| Data editing |
| --- |
| bayesboot [1] |
| DataExplorer [2] |
| dlookr [3] |
| dplyr [4] |
| caret [5] |
| ggpubr [6] |
| lubridate [7] |
| missRanger[8] |
| plotly [9] |
| plyr [10] |
| tidyr [11] |
| tidyverse [12] |
| visdat [13] |
| Analyses |
| ggplot2 [14] |
| randomForest [15] |
| rfPermute [16] |
| vip [17] |

**References**

1. Baath R. bayesboot: an implementation of Rubin’s (1981) bayesian bootstrap. R package version 0.2.2. 2018.

2. Cui B. DataExplorer: automate data exploration and treatment. R package version 0.8.2. 2020.

3. Ryu C. dlookr: Tools for data diagnosis, exploration, transformation. 2022. R package version 0.6.0

4. Wickham H, Francois R, Henry L, Müller K. dplyr: A grammar of data manipulation. R package version 1.0.7. 2021.

5. Kuhn M. caret: classification and regression training. R package version 6.0-93. 2022.

6. Kassambara A. ggpubr: ggplot2' based publication ready plots. R package version 0.4.0. 2020.

7. Grolemund G, Wickham H. Dates and times made easy with lubridate. J Stat Softw. 2011;40:1-25.

8. Mayer M. missRanger: Fast Imputation of Missing Values. 2021. R package version 2.1.3.

9. Sievert C. Interactive web-based data visualization with R, plotly, and shiny: Chapman and Hall/CRC; 2020.

10. Wickham H. The split-apply-combine strategy for data analysis. J Stat Softw. 2011;40:1-29.

11. Wickham H, Girlich M. tidyr: Tidy messy data. 2022. R package version 1.2.0.

12. Wickham H, Averick M, Bryan J, Chang W, D’Agostino McGowan L, et al. Welcome to the tidyverse. J Open Source Softw. 2019;4:1686.

13. Tierney N. visdat: Visualising whole data frames. J Open Source Softw. 2017;2:355.

14. Wickham H. ggplot2: elegant graphics for data analysis: Springer-Verlag, New York, USA; 2016.

15. Liaw A, Wiener M. Classification and regression by randomForest. R News. 2002;2:18-22.

16. Archer E. Estimate permutation p-values for random forest importance metrics. R package version 2.5.1. 2022.

17. Greenwell B, Boehmke b. Variable importance plots-an introduction to the vip package. The R Journal. 2020;12:343-66.
